# Validation and application of sub-2 μm core–shell UHPLC–UV–ESI–Orbitrap MS for identification and quantification of β-carotene and selected cleavage products with preceding solid-phase extraction

**DOI:** 10.1007/s00216-014-7725-8

**Published:** 2014-03-21

**Authors:** G. Martano, E. Bojaxhi, I. C. Forstenlehner, C. G. Huber, N. Bresgen, P. M. Eckl, H. Stutz

**Affiliations:** 1 Division of Chemistry and Bioanalytics, Department of Molecular Biology, University of Salzburg, 5020 Salzburg, Austria; 2 Division of Genetics, Department of Cell Biology, University of Salzburg, 5020 Salzburg, Austria

**Keywords:** β-Carotene, Apo-carotenals, Cell culture media, Solid-phase extraction, Core–shell particles, UHPLC-UV-MS, Validation

## Abstract

A validated ultrahigh-performance liquid chromatography method using 1.7 μm core–shell particles is presented for the identification and quantification of β-carotene (BC) and related cleavage products (CPs) in primary cell culture media. Besides BC, apo-4′-, apo-8′-, apo-10′-, and apo-12′-carotenals, as well as 5,6-epoxy-β-carotene, were selected as target analytes. Detection was performed via an 80-Hz diode array detector and an electrospray ionization–linear quadrupole ion trap–Orbitrap XL mass spectrometer, both hyphenated in series. Total analysis time was below 6 min with peak widths <12 s. Addition of trifluoroacetic acid and tetrahydrofuran to the mobile phase allowed for the mass spectrometric detection of BC and related CPs and reduced peak tailing due to improved solubility of hydrophobic analytes. Intra-day and inter-day precision for UV and mass spectrometric detection were ≤1.5 % for retention times and ≤5.1 % for peak areas. Instrumental linearity was confirmed by Mandel’s fitting test between 0.25 (or 1.00 μg/mL) and 5.00 μg/mL for UV detection. The higher sensitivity of mass spectrometric detection allowed for the coverage of three concentration domains between 0.025 and 5.00 μg/mL in linearity testing. Homoscedasticity was confirmed between 0.10 and 5.00 μg/mL for Orbitrap XL MS. The limits of quantification were between 52.6 and 889.4 ng/mL for UV detection and between 19.3 and 102.4 ng/L for mass spectrometric detection. Offline solid-phase extraction from culture media fortified with BC and CPs provided intra- and inter-day recoveries between 65.8 and 102.4 % with coefficients of variation ≤6.2 %. Primary rat hepatocyte cultures treated with BC and subjected to different oxidative stress conditions contained 5,6-epoxy-BC and apo-4′-carotenal besides residual BC. Apparently, 5,6-epoxy-BC was formed in the medium via autoxidation of BC by ambient oxygen.

## Introduction

Carotenoids and particularly β-carotene (BC)—a precursor of vitamin A—have been assigned beneficial health effects [1] including a prominent role in the prevention of cataract, senile macular degeneration, cardiovascular diseases, neurodegenerative diseases, cancer, and in cancer therapy [2, 3]. Several studies have provided evidence for anti-oxidative effects of BC by quenching singlet oxygen and scavenging peroxy radicals [4, 5]. Thus, BC has been applied comprehensively in micro-supplementation [6]. Besides, BC and its degradation product apo-8′-carotenal were frequently applied as food and beverage colorants *E 160 a* and *E 160 e* [7].

Nonetheless, certain frame conditions trigger pro-oxidative and pro-carcinogenic effects of carotenoids [8, 9]. High oxygen pressure, pronounced oxidative stress, and BC administration at elevated doses apparently promote adverse effects [4, 10]. Two large-scale human intervention trials, i.e., the ATBC [11] and the Carotene and Retinol Efficacy (CARET) [12, 13] studies, have addressed this paradox by oral long-term administration of 20–30 mg BC/day to human probands. Test persons comprised inter alia cigarette smokers and workers exposed to asbestos. These groups showed a 16–28 % increased lung cancer incidence with higher mortality risk when compared to control groups which requested the termination of the CARET trial ahead of schedule [12, 13]. Other BC supplementation studies, such as the Physician Health Study (PHS) [14] and the Linxian Trial [15, 16], revealed no effect on the health state or demonstrated indeed reduced cancer-related mortality [5, 14]. Different dietary basis uptake of BC and pre-existing tumors might explain divergent study results [5, 16].

Adverse effects in humans have been mimicked in animal models sharing physiological similarity in BC adsorption and tissue metabolism, i.e., ferrets [2, 16]. Thereby, BC supplementation at pharmacological doses was combined with exposure to cigarette smoke [17]. The high number of free radicals prevailing in cigarette smoke promotes the formation of BC oxidation products (= cleavage products (CPs)) [18]. Moreover, CPs, such as apo-10′- [19] and apo-14′-carotenal, have also promoted the binding of benzo[*a*]pyrene to DNA [16]. Apparently, the described BC paradox was not evoked by BC per se but by its degradation products (CPs) generated by eccentric cleavage as stated elsewhere [16, 18, 20]. This has been corroborated by recent studies on primary rat hepatocytes treated with apo-8′-carotenal and CP mixtures [21, 22]. Non-volatile CPs, e.g., apo-carotenals, epoxides, and carbonyls, are generated by hypochloric acid (HClO) that is formed by myeloperoxidase secreted from activated phagocytes [23]. The reaction mechanism between BC and HClO has been mimicked in vitro by Handelman et al. [24] and revealed a comprehensive set of CPs. The proposed eccentric degradation products [25] are also present to a minor extent in intestinal mucosa cells and hepatic cells due to the presence of β-carotene-9′,10′-monooxygenase (BCO 2) [26, 27].

Carotenoids have preferably been analyzed by high-performance liquid chromatography (HPLC) with porous C_18_ [28, 29] and C_30_ [7, 30–33] particles employing UV [19, 29, 31, 33, 34], quadrupole mass spectrometric (MS) [19, 35], and ion-trap MS detection [32]. Contrary to carotenoid mixtures, CPs, such as apo-carotenals, have scarcely been addressed [24, 36, 28, 37] and yet less frequently in cells or cell cultures [34]. HPLC separations with totally porous particles suffer from extended analysis times, i.e., 20–83 min for carotenoid mixtures [6, 7, 30, 31, 33] and BC with related CPs [3, 24]. Ultrahigh-performance LC (UHPLC) [38] offers improved selectivity and accelerated separations [39, 40]. On the other hand, superficially porous particles with a solid, impenetrable core provide reduced back-pressure [41] and allow for excellent reduced plate height values down to 1.1 [39, 42, 43].

Linear quadrupole ion trap (LTQ)–Orbitrap MS with full scan option provides the possibility for a mid-term untargeted strategy for high sensitivity, mass accuracy in low parts per million range [44], and a mass resolution of >100,000 (FWHM) [45]. Consistently, the hyphenation of UHPLC to linear ion trap–Orbitrap MS represents a highly promising combination for UHPLC–MS analysis of carotenoids.

The current work aims to develop and validate an UHPLC–diode array detection (DAD)–electrospray ionization (ESI)–Orbitrap MS method for BC and related CPs selected from previously described degradation profiles [24, 25] applying sub-2 μm core–shell particles with preceding offline solid-phase extraction (SPE). The combination with offline SPE intends to further reduce interferences [46]. Thereby, the SPE strategy recently developed for volatile CPs [47] should be adopted to allow for a simultaneous extraction of volatile and non-volatile CPs, and BC with optional splitting of the eluate for final GC–EI–MS [47] and UHPLC–DAD–ESI–Orbitrap MS analyses. This combined extraction step for CPs of highly variant properties provides an innovation in the analysis of cell culture media. Analyte identification refers to a combination of several decision parameters, i.e., *t*_R_, UV spectra, accurate masses, and related natural isotopic patterns.

## Materials and methods

### Reagents and chemicals

**Fig. 1:**
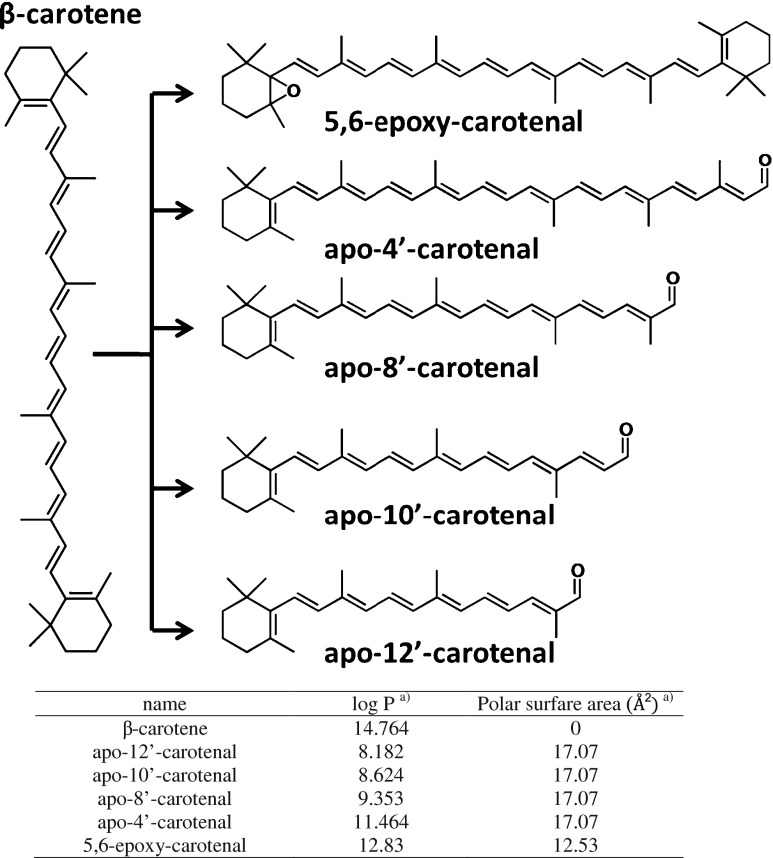
Degradation scheme of *all*-*trans* BC (by HClO according to [25]) with structures and hydrophobicity values of related non-volatile target cleavage products

*All*-*trans*-β-carotene (BC; purity ≥97.0 %), *all*-*trans*-β-apo-8′-carotenal (apo-8′; purity ≥96.0 %), methanol (purity ≥99.9 %), *n*-hexane (in UniSolv quality), trifluoroacetic acid (TFA; purity ≥99.5 %), H_2_O_2_ (in semi-conductor quality), Fe(II)-lactate (purum ≥98 %), minimum essential medium Eagle (MEM), and Tween 20 (for molecular biology) were all obtained from Sigma-Aldrich/Fluka (St. Louis, MO, USA). Methylisoeugenol (purity >98 %) was obtained from SAFC (St. Louis, MO, USA) and used as internal standard (IS) as outlined previously [47]. β-Apo-4′-carotenal (apo-4′), β-apo-10′-carotenal (apo-10′), β-apo-12′-carotenal (apo-12′), and 5,6-epoxy-carotenal (5,6-epoxy-BC) were received as lyophilized powder from CaroteneNature GmbH (Lupsingen, Switzerland) at 92–99 % purity and stored light protected at −20 °C. Analyte structures are given in Fig. 1. Tetrahydrofuran (THF; anhydrous and without stabilizer) was from Merck (Darmstadt, Germany). Acetonitrile (ACN; Chromasolv, purity ≥99.8 %) was purchased from Promochem (Wesel, Germany). 2,3-Dimethoxy-1,4-naphthoquinone (DMNQ; ≥99 %) was obtained from Enzo Life Sciences (Lausen, Switzerland). Ultrapure water was prepared by a Milli-Q Plus 185 system (Millipore S.A., Molsheim, France). Nitrogen in 5.0 quality was obtained from SIAD GmbH (St. Pantaleon, Austria).

### Ultrahigh-performance liquid chromatography–diode array detection–electrospray ionization–Orbitrap mass spectrometry

**Fig. 2:**
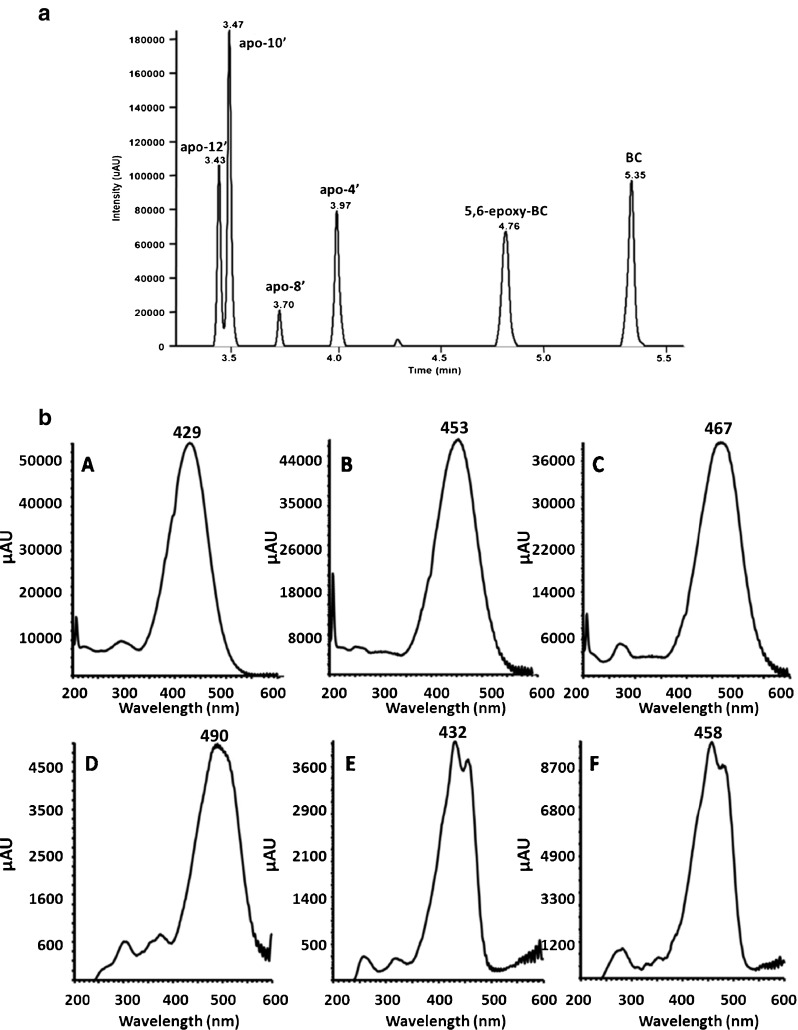
**a** UHPLC chromatogram acquired at 460 nm for a standard solution containing BC and CPs at 1.0 μg/mL. **b** Corresponding UV spectra of: *A* apo-12′-carotenal, *B* apo-10′-carotenal, *C* apo-8′-carotenal, *D* apo-4′-carotenal, *E* 5,6-epoxy-carotenal, and *F* β-carotene

UHPLC analyses were performed with an Accela System, equipped with an Accela 1250 pump, an Accela 80-Hz diode array detector with a wavelength range from 190 to 600 nm and equipped with a 2-μl LightPipe flow cell (all from Thermo Fisher Scientific, Palo Alto, CA, USA), and an LC PAL DLW Option Autosampler (from CTC Analytics AG, Zwingen, Switzerland). Acquisition of UV data was performed at the respective absorbance maximum of either analyte (Table 1; Fig. 2). Due to the narrow peak widths of 2–10 s, a data sampling rate of 40 Hz was selected. The UHPLC system was equipped with a 2-μl injection loop and a column oven 200 (from Thermo Fisher Scientific), which was set to 50 °C. During the injection, the entire loop was filled. For chromatographic separations, a Kinetex® C18 column (100 × 2.1 mm ID, 1.7 μm, 100 Å; Phenomenex, Torrance, CA, USA) with core–shell particles was operated at a flow rate of 500 μl/min. Separation was conducted by a ternary gradient including (A) ultrapure water with 0.02 % (*v*/*v*) TFA, (B) ACN with 0.02 % (*v*/*v*) TFA, and (C) 50 % (*v*/*v*) THF with 0.02 % (*v*/*v*) TFA (in ACN) employing the following gradient program: 0.00 min—60 % A/20 % B/20 % C, 0.50 min—60 % A/20 % B/20 % C, 2.00 min—10 % A/44 % B/46 % C, 5.00 min—10 % A/0 % B/90 % C, 7.00 min—10 % A/0 % B/90 % C, 7.50 min—60 % A/20 % B/20 % C, and 9.00 min—60 % A/20 % B/20 % C. The UHPLC back-pressure was between 750 and 950 bar during the separation.

**Table 1:** Survey of retention times and detection wavelengths (according to the absorbance maxima derived from Fig. 2b) as well as theoretical and recorded *m*/*z* for target CPs and BC with optimized UHPLC–DAD–ESI–Orbitrap MS

	Apo-12′-carotenal	Apo-10′-carotenal	Apo-8′-carotenal	Apo-4′-carotenal	5,6-Epoxy-carotenal	β-Carotene
Retention time (min)	3.43	3.47	3.70	3.97	4.76	5.35
Wavelengths (nm)	425	450	470	490	430	460
*m*/*z*_theor_	351.26824	377.28389	417.31519	483.36214	553.44039	536.43765
*m*/*z*_detect_	351.26794	377.28400	417.31375	483.36129	553.44067	536.43713
ppm	<1.5	<1.5	<3.5	<2.0	<1.0	<2.0

Due to its high mass accuracy, mass stability, detection sensitivity, and analyte specificity, Orbitrap MS was preferred over time-of-flight MS or triple–quadrupole MS, allowing the highly confident identification of the analytes extracted from biological samples. Mass spectrometric measurements were performed by an LTQ–Orbitrap XL mass spectrometer which was hyphenated by the Ion Max ESI ion source (all from Thermo Fisher Scientific) to the UHPLC system. Tuning of the system was done in the automatic tune mode by means of a 100-μg/mL apo-8′ solution prepared in 50 % (*v*/*v*) THF–50 % (*v*/*v*) ACN for the monoisotopic mass of [apo-8′ + H^+^] at *m*/*z* 417.3, by adding the tuning solution to the column effluent via a T-splitter. The optimized MS parameters included a heated capillary temperature of 250 °C; sheath gas and auxiliary gas flow of 30 and 15 arb. units, respectively; a source voltage of +3.0 kV; a capillary voltage of 46.00 V; and a tube lens voltage of 35.00 V. Calibration of the system was performed according to the protocol of the manufacturer. Data acquisition in single ion monitoring (SIM) mode with the LTQ–Orbitrap XL mass spectrometer addressed theoretical masses of analytes with a tolerance window of ±0.5 *m*/*z*. By reason of the targeted approach in mass spectrometry, SIM mode is preferred over full scan. Due to the analysis of cell culture media, we can expect a number of other compounds present in the sample apart from the target analytes. The SIM mode enables to selectively address the exact molecular masses of our target analytes which increases the clarity of the obtained chromatograms and aids in data evaluation. Moreover, the selective tuning for defined masses enhances the transmission thus improving sensitivity. For the extracted ion current chromatograms (EICC) corresponding masses were selected with a reduced mass width of ±0.05 *m*/*z*. To address target analytes, masses were therefore set to *m*/*z* 351.26 and 377.28 for the first 3.60 min, then switched to *m*/*z* 417.31 from 3.60 to 3.85 min, to *m*/*z* 483.36 up to 4.20 min, to *m*/*z* 553.44 up to 5.00 min, and finally to *m*/*z* 536.43 from 5.00 min to the end of the run.

### Standard solution for UHPLC–DAD–ESI–Orbitrap MS instrumental validation

Single standard solutions of BC, of the selected apo-carotenals, and of 5,6-epoxy-BC were prepared gravimetrically at nominal 1.0 mg/mL in 50 % (*v*/*v*) ACN–50 % (*v*/*v*) THF. These single standard solutions were used to prepare 1.0 mL of a composite standard stock solution with 100 μg/mL for either compound by mixing them in an appropriate ratio and adjusting the final volume with 50 % (*v*/*v*) ACN–50 % (*v*/*v*) THF. This composite stock solution was light-protected and stored under nitrogen at +4 °C. From this composite stock solution, working standard solutions for calibration and for linearity testing were freshly prepared by dilution to appropriate concentrations with ACN and THF (50:50 % (*v*/*v*)) immediately prior to their injection into the UHPLC–DAD–ESI–Orbitrap MS system. Therefore, different concentration ranges, i.e., 0.025–0.10, 0.10–1.0, and 1.0–5.0 μg/mL, were considered.

### Standard solutions for validation of SPE offline coupled to UHPLC–DAD–ESI–Orbitrap MS

A composite standard stock solution containing BC and all target CPs at 200 μg/mL, respectively, was prepared in an aqueous 9.5 mmol/L Tween 20 solution. Methylisoeugenol, which was previously employed as IS to correct for differences in the SPE efficacy [47], was also applied for BC and non-volatile CPs and included at 200 μg/mL in the composite standard. This composite stock solution was diluted with MEM at ratios of either 1:200 (*v*/*v*) or 1:400 (*v*/*v*) to give working solutions of 1.0 or 0.5 μg/mL for BC and individual CPs, respectively. Working solutions were prepared immediately prior to their application in SPE and employed for the determination of SPE recoveries at these concentrations as well as in the inter- and intra-day assays for the selected SPE adsorbent.

### Solid-phase extraction

Strata Phenyl 500 mg/3 mL SPE columns were obtained from Phenomenex and applied in the SPE optimization for BC and apo-carotenals. The SPE procedure is based on a recently published SPE method for volatile cleavage products of β-carotene [47]. Briefly, SPE columns were conditioned with 3 mL methanol followed by 3 mL ultrapure water before 1.0 mL of blanks, of the respective MEM working solution—spiked either with β-carotene and the internal standard or β-carotene, related cleavage products, and the internal standard—or of the cell culture medium after incubation was loaded. The column was then washed with 2 mL ultrapure water. Elution of β-carotene, non-volatile cleavage products, and methylisoeugenol (IS) was done with 2.0 mL 10 % (*v*/*v*) THF in *n*-hexane at a flow rate ≥2 mL/min to comply with the SPE requirements of volatile cleavage products measurements by GC–EI–MS. The eluate was cooled to −20 °C to facilitate the separation of the organic and aqueous phase. The hydrophobic fraction of the eluate was then collected [47]. The implementation of this SPE strategy offers a simultaneous extraction of volatile and non-volatile CPs for future analyses with subsequent equal splitting of the 2-mL eluate for GC–EI–MS and UHPLC–DAD–ESI–Orbitrap MS, respectively. When analyzing only BC and non-volatile CPs, the entire eluate can alternatively be transferred to a Kuderna Danish micro-evaporator system and evaporated to dryness under a gentle stream of nitrogen. Residues were reconstituted in 1.0 mL 50 % (*v*/*v*) ACN–50 % (*v*/*v*) THF and then injected directly into the UHPLC–DAD–ESI–Orbitrap MS system.

### Solutions for treatment of cell cultures

Stock solutions of BC for controls and cell treatment were prepared at 10 mmol/L in 9.5 mmol/L aqueous Tween 20 solution. This stock solution was diluted 1:100 (*v*/*v*) with MEM to provide a final concentration of 100 μmol/L BC. An appropriate volume of this diluted BC treatment solution was then spiked to the cell culture medium to make up a further 1:10 dilution. Consistently, the final concentration in the cell culture was 10 μmol/L BC including also 9.5 μmol/L Tween 20. Prior to the addition to cell cultures, the spiked culture medium is mixed to assure a homogenous solution. Cytotoxic effects in cell cultures of primary hepatocytes of female Fischer 344 rats were absent at the tested Tween 20 concentration in MEM. DMNQ, H_2_O_2_, and iron lactate stock solutions were all prepared at 1.0 mmol/L in serum-free MEM, respectively.

### Primary hepatocyte cultures

Primary hepatocytes were chosen as a model system because the culture medium is not supplemented with serum (regularly 10–20 %) and contains only proteins excreted by the cells [48], thus facilitating the chemical analysis. Primary parenchymal hepatocytes were prepared from female Fischer 344 rats according to the protocols given elsewhere [48, 49]. Isolated primary hepatocytes were cultivated in 5 mL serum-free MEM supplemented with non-essential amino acids, pyruvate (1 mmol/L), aspartate (0.20 mmol/L), serine (0.20 mmol/L), and penicillin (100 U)/streptomycin (100 μg/mL) in collagen-coated 60-mm-diameter plastic culture dishes for 21 h with change of the culture medium after the first 3 h. After these 21 h, incubation of cells with the respective treatment solutions was performed directly without any further change of medium for 3 h. Incubation under control conditions was done at 37 °C, 5 % CO_2_, and 95 % relative humidity without oxidative stress treatment applying either culture medium alone (i.e., deficient of Tween 20 and BC) or fortified with appropriate volumes of DMNQ, H_2_O_2_, or Fe^++^ stock solutions. Fortified culture media were prepared immediately prior to their application, which was done by replacing the medium after 21 h incubation. In case of treatment, primary hepatocytes were incubated with 10 μmol/L BC and concurrent oxidative stress.

### Cell treatment with BC under oxidative stress

Different approaches were applied to mimic the oxidative stress of the risk groups in the chemoprevention trials employing the validated SPE UHPLC–DAD–ESI–Orbitrap MS method to culture media. The tested treatment solutions for primary hepatocytes address in vitro degradation of BC to non-volatile CPs over the incubation interval, subsequent to BC spiking to the cell culture medium. In case of treatment under different prooxidant conditions, primary hepatocytes were incubated for 3 h with 10 μmol/L BC in the presence of either (i) 40 μmol/L DMNQ, (ii) 10 μmol/L H_2_O_2_, or (iii) a combination of 10 μmol/L H_2_O_2_ and 10 μmol/L Fe(II)lactate—the latter subsequently termed “Fenton condition” [50]. Cultures exposed to these regimens without addition of BC served as references for oxidative conditions. For chemical analysis, the culture supernatants were collected immediately after the 3-h treatment period and subjected to SPE. For cultures treated with BC and subsequent oxidative stress, hepatocytes from two different animals were prepared on different days. Hepatocytes of each rat were cultivated in three separate Petri dishes, respectively. For either Petri dish, one SPE was performed and the final eluate analyzed in triplicate.

### Statistical treatment

Data were statistically evaluated by means of the SPSS Statistical Software Package version 16 applying appropriate test procedures, e.g., one-way analysis of variance (ANOVA), Levene’s test, and independent *t* test. Mandel’s fitting test (MFT) is not included as standard SPSS operation and was thus established via the SPSS syntax function. Details of the calculation procedure have been outlined recently [47] and are briefly recalled in the respective sections. Results were considered significant at *p* < 0.05.

## Result and discussion

The selection of non-volatile target analytes, i.e., apo-carotenals and 5,6-epoxy-BC, was based on the constituent profiles of CPs previously described for the in vitro cleavage of BC with HClO [24] and for activated neutrophils [10] and other cells, such as pneumocytes [34], when incubated with BC.

### UHPLC with core–shell columns

Currently, UHPLC separations have been merely published for carotenoids, retinol and tocopherol [33, 51], but not for BC and related CPs, i.e., apo-carotenals. In comparison to previously published HPLC separations for apo-carotenals which required 30–40 min total analysis time [52], the developed UHPLC separation was performed in less than 6 min with peak widths at base of analyte peaks smaller than 12 s. The gradient composition and programming were essential to achieve narrow peak widths and avoid precipitation as well as trapping of highly hydrophobic analytes on the column. Therefore, a ternary gradient composed of ultrapure water, ACN, and THF was crucial for dissolving moderately and highly hydrophobic analytes in a moderate hydrophilic solution.

TFA has been added both to reduce secondary ionic interactions by shielding residual silanol groups [53] and reduce the pH in the mobile phase to promote ionization in positive mode. During the optimization of the UHPLC separation, severe tailing of the BC peak due to a restricted solubility [54] was an issue of concern (data not shown). Therefore, the THF content in the mobile phase was stepwise increased within the gradient up to final 45 % (*v*/*v*) immediately before BC was passing the detector. This improved peak shape and symmetry substantially and is in accordance with recent strategies [19]. Although a minor tailing of the BC peak persisted under the selected conditions (Fig. 2a), an increase beyond the selected THF content showed no further improvement (data not shown). UV spectra for BC and related CPs recorded under the selected UHPLC conditions are depicted in Fig. 2b together with related absorbance maxima (Table 1). These wavelengths were also selected for detection of analytes and validation of UHPLC–DAD. The maximum at 458 nm and the side maximum at 487 nm for BC correspond to previous data [36] despite a minor shift which is related to the different compositions of the mobile phase [54]. Apo-carotenals showed an increase in their respective absorbance maximum in the order of apo-12′ to apo-4′ due to the increase in the polyene chain length [54], as described elsewhere [36, 28, 55]. As expected, the elution order of BC and related CPs is governed by their hydrophobicity (Figs. 1 and 2a).

### LTQ–Orbitrap MS

**Fig. 3:**
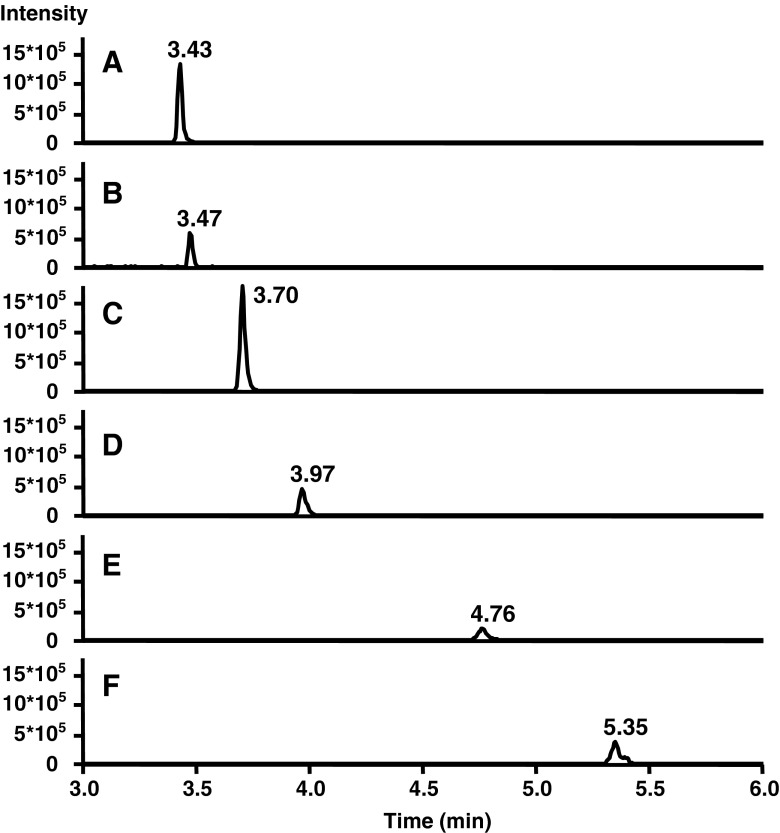
Extracted ion current chromatograms of a standard solution containing BC and target CPs at 1.0 μg/L. Identity of peaks: *A* apo-12′-carotenal, *B* apo-10′-carotenal, *C* apo-8′-carotenal, *D* apo-4′-carotenal, *E* 5,6-epoxy-BC, and *F* β-carotene

**Fig. 4:**
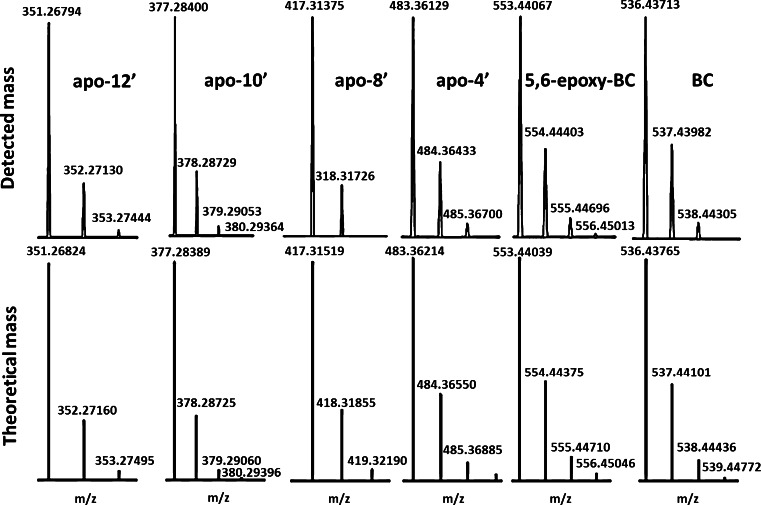
Detected masses and isotope distribution for BC and target apo-carotenals including a comparison with theoretical masses and theoretical isotope distribution by means of the provided software option

The lack of protonation sites in BC and other carotenes makes their ionization in ESI–MS inefficient if not taking appropriate means to ameliorate the situation. However, this does not refer to the CPs which can be protonated (see structures in Fig. 1). Ionization of BC has been realized either by post-column addition of oxidizing agents, e.g., halogenated solvents [56], or of silver salts [32, 57]. In the current case, ionization of BC is effected upon electrochemical oxidation [58] with formation of radical ions (M^•+^) [56, 57]. Moreover, it has been shown that electrochemical oxidation is supported under acidic condition [56]. Contrary to BC, all target CPs are detected as [M + H]^+^ under the selected conditions. EICCs of a standard solution are depicted in Fig. 3. In addition, detected masses and isotope patterns for BC and target apo-carotenals are included and compared with their theoretical masses and simulated isotope patterns (Fig. 4) which were used for formula confirmation.

### Solid-phase extraction in sample preparation

The concept of the presented SPE approach pursues the strategy of a simultaneous extraction of physicochemically divergent analytes, i.e., BC, non-volatile, and volatile CPs. Thus, the UHPLC–DAD–ESI–Orbitrap MS method for non-volatile CPs should share the SPE procedure with GC–EI–MS for volatile CPs [47]. This strategy ensures identical adsorption and elution conditions for all target analytes and reduces variations in the SPE performance for individual analyte classes within the same sample. However, since GC–EI–MS and UHPLC–DAD–ESI–Orbitrap MS are applied as final analysis steps, a splitting of the eluate and change to solvents compatible with the final separation and detection step are required. As in GC–EI–MS, methylisoeugenol was applied as an IS to correct quantification in UHPLC–DAD–ESI–LTQ–Orbitrap MS.

### Method validation

#### Instrument validation of UHPLC–DAD–ESI–Orbitrap MS

The developed UHPLC–DAD–ESI–Orbitrap MS method was subject of a single-laboratory validation according to the IUPAC and the relevant ICH guideline Q2(R1) [59, 60]. Instrumental basis validation addressed intra-day and inter-day precision of retention times (*t*_R_) and peak areas, linearity testing for up to three concentration ranges, homoscedasticity testing, and determination of limit of detection (LOD) and limit of quantification (LOQ) for UV and mass spectrometric detection, respectively. Results are given in Tables 2, 3, and 4. Peak areas were corrected by IS for determination of precision and linearity testing. All data were based on composite standard solutions containing BC and CPs dissolved in 50 % (*v*/*v*) ACN–50 % (*v*/*v*) THF (including also the IS). Representative chromatograms with UV and MS detection of BC and CPs are given in Figs. 2 and 3.

**Table 2:** Validation parameters for calibration, linearity, and homoscedasticity testing for UHPLC–UV

Analytes	Concentration range (μg/mL)	Linear regression of instrument *y* = *bx* + *a*				Linearity testing MFT^b^
		Slope (= *b*)	Intercept (= *a*)	*p* (for *a*)^a^	*R* ^2^	
Apo-12′ (*n* = 24)	0.25–5.00	69,623.67	−488.18	>0.05	0.9995	Passed
Apo-10′ (*n* = 24)	0.25–5.00	57,220.16	−1,229.99	>0.05	0.9993	Passed
Apo-8′ (*n* = 24)	0.25–5.00	48,495.34	−991.18	>0.05	0.9996	Passed
Apo-4′ (*n* = 15)	1.00–5.00	30,093.66	−2,689.44	<0.05*	0.9989	Passed
5,6-Epoxy-BC (*n* = 15)	1.00–5.00	42,282.97	−2,090.50	>0.05	0.9992	Passed
BC (*n* = 15)	1.00–5.00	28,645.50	−4,474.17	<0.05*	0.9966	Passed

Significance of slope, i.e., difference from 0, was proven at a confidence level of 0.95 by ANOVA for all regression equations

*p < 0.05 refers to a significant difference of the intercept from 0

^a^Refers to the significance of coefficient *a* (= intercept) defined in the regression analysis

^b^Mandel’s fitting test at a confidence level of 99.0 %

**Table 3:** Validation parameters for calibration, linearity, and homoscedasticity testing for UHPLC–ESI–Orbitrap

Analyte		Concentration range (μg/mL)	*y* = *bx* + *a*				Linearity testing MFT^b^	Homoscedasticity^c^
			Slope (= *b*)	Intercept (= *a*)	*p* (for *a*)^a^	*R* ^2^		
Apo-12′	*n* = 36	0.025–5.00	2,885,781.45	484,003.92	<0.05*	0.9930	All passed	
	*n* = 12	0.025–0.10	5,935,002.67	−60,738.67		0.9842		
	*n* = 15	0.10–1.00	3,741,876.42	244,501.59		0.9886		4.97
	*n* = 15	1.00–5.00	2,646,606.57	1,333,099.77		0.9948		
Apo-10′	*n* = 36	0.025–5.00	3,051,117.73	520,893.39	<0.05*	0.9925	All passed	
	*n* = 12	0.025–0.10	6,789,585.33	−110,993.17		0.9844		
	*n* = 15	0.10–1.00	3,989,556.79	299,099.27		0.9870		3.28
	*n* = 15	1.00–5.00	2,823,405.40	1,319,197.93		0.9937		
Apo-8′	*n* = 36	0.025–5.00	2,504,361.99	1,125,333.03	<0.05*	0.9518	Passed	
	*n* = 12	0.025–0.10	8,080,462.67	−65,880,50		0.9949	Passed	
	*n* = 15	0.10–1.00	4,650,150.04	415,127.24		0.9890	Failed	2.29
	*n* = 15	1.00–5.00	1,837,987.17	3,513,752.77		0.9784	Passed	
Apo-4′	*n* = 36	0.025–5.00	2,000,767.56	1,219,207.53	<0.05*	0.9111	All passed	
	*n* = 12	0.025–0.10	7,579.605.33	−105,700.17		0.9889		
	*n* = 15	0.10–1.00	4,572,789.19	401,076.76		0.9815		2.67
	*n* = 15	1.00–5.00	1,274,599.87	3,811,724.60		0.9722		
5,6-Epoxy-BC	*n* = 36	0.025–5.00	1,895,446.45	1,235,499.33	<0.05*	0.8817	All passed	
	*n* = 12	0.025–0.10	7,489,329.33	−122,794.33		0.9848		
	*n* = 15	0.10–1.00	4,547,332.80	344,518.48		0.9776		3.36
	*n* = 15	1.00–5.00	1,090,017.30	4,124,605.30		0.8653		
BC	*n* = 36	0.025–5.00	2,383,560.31	1,172,578.04	<0.05*	0.8996	All passed	
	*n* = 12	0.025–0.10	5,744,781.33	−110,962.83	<0.05*	0.9707		
	*n* = 15	0.10–1.00	5,558,415.89	−40,412.59	>0.05	0.9825		3.95
	*n* = 15	1.00–5.00	1,394,933.97	4,744,144.23	<0.05*	0.8947		

Significance of slope, i.e., difference from 0 was proven at a confidence level of 0.95 by ANOVA for all regression equations

*Refers to a significant difference (*p* < 0.05) of the intercept from 0

^a^Refers to the significance of coefficient *a* (= intercept) defined in the regression analysis

^b^Mandel’s fitting test at a confidence level of 99.0 %

^c^Homoscedasticity was tested within 0.1 and 5.0 μg/mL (*n* = 10, respectively)

**Table 4:** Limit of detection and limit of quantification for BC and CPs

		Apo-12′	Apo-10′	Apo-8′	Apo-4′	5,6-Epoxy-BC	BC
LOD (ng/mL)	UV	24.6	17.3	30.0	293.5	221.2	231.4
	MS	0.0109	0.034	0.0064	0.0128	0.0151	0.0094
LOQ (ng/mL)	UV	74.6	52.6	87.1	889.4	639.9	741.3
	MS	0.0329	0.1024	0.0193	0.0387	0.0458	0.0285

##### Precision

As part of the instrumental basis validation, intra- and inter-day precision of *t*_R_ and corrected peak areas were determined for UV and MS detection. Therefore, a 2.0-μg/L standard solution including the IS was injected repetitively. For calculation of the intra-day precision, this standard solution was injected five times, whereas the inter-day precision was based on three replicate injections on five consecutive days. Coefficients of variation (CV) for *t*_R_ are given only for mass spectrometric data, since due to its higher spatial distance from the injection site, this detection system is expected to give higher CVs of *t*_R_. Both intra- and inter-day precision of *t*_R_ were ≤1.5 % (corresponding to deviations of less than 2 s). Precision of corrected peak areas was ≤4.1 % for intra-day and ≤5.1 % for inter-day measurements for UV and mass spectrometric detection, respectively.

##### Linearity

Response linearity of DAD and Orbitrap MS for BC and related CPs was tested with both detection systems hyphenated in series to UHPLC. Two ranges between 0.25 and 5.00 μg/mL were covered for UV. However, due to the higher sensitivity of Orbitrap MS in comparison to UV detection, another low concentration domain was included to extend quantification to lower concentrations of CPs. Consistently, three concentration ranges between 0.025 and 5.00 μg/mL were tested with Orbitrap MS. Each calibration region encompassed four to five different concentrations evenly distributed over the respectively addressed domain. Regression equations refer to the least square approach and consider peak areas corrected by IS. In the case non-linearity is revealed by MFT over the entire concentration range, a restriction to individual concentration ranges based on the respective signal intensity will offer improved quantification.

##### DAD

The tested concentration ranges for UV covered 0.25–5.00 μg/mL. Analyte signals were acquired at their respective wavelengths as given in Table 1. The highest concentration tested, i.e., 5.00 μg/mL, refers to the BC concentration intended for subsequent application in primary cell cultures to reveal possible genotoxic effects with concomitant oxidative stress. The significance of intercepts was tested by means of an ANOVA approach offered by SPSS. Additionally, ANOVA was also applied to evaluate the linear regression model, i.e., the significance of the slope, which was confirmed in either case (data not shown). Furthermore, linearity was tested by means of MFT [61]. The coefficient of determination *R* ^2^ which indicates the correlation between the applied concentration and the measured signal, i.e., peak area, but does not represent a measure for linearity [62]—as frequently stated erroneously—is given as well. All data are surveyed in Table 2. However, due to the higher LOQ of apo-4′, 5,6-epoxy-BC, and BC that were >0.50 μg/mL (see Table 4), linearity was only considered between 1.00 and 5.00 μg/mL for these CPs (see Table 2).

The MFT indicated no improved fitting for a second order regression model for either tested concentration domain. Even when both tested concentration regions were combined in case of apo-12′-, apo-10′-, and apo-8′, covering the entire domain of 0.25–5.00 μg/mL, calculated *F* values derived by MFT proved no significance (see Table 2). This implies linearity over the entire concentration range tested in UV.

##### Orbitrap MS

Due to the enhanced sensitivity of Orbitrap MS in comparison to the previously addressed UV detection, the tested concentration range was extended to lower concentrations and divided into three consecutive domains, i.e., 0.025–0.10, 0.10–1.00, and 1.00–5.00 μg/mL which allows for a quantification of CPs and BC in considerably lower concentrations than with UV detection. Calculation of the significance of the intercept, as well as linearity testing with MFT and the evaluation of the linear regression model with ANOVA, was performed as outlined in the previous paragraph. Measured peak areas refer to extracted ions for the monoisotopic masses of BC and target CPs acquired in SIM mode (see Table 1) with a mass window of ±0.05 *m*/*z*.

With increasing concentrations, results of Orbitrap MS indicated a continuous reduction in the slope (Table 3). However, the statistical evaluation by MFT indicated significant deviations from linearity only for apo-8′. In parallel, *R* ^2^ are also considerably reduced in comparison to corresponding UV data, which additionally points to reduced correlations. This is particularly true for the highest concentrations between 1.00 and 5.00 μg/mL, with the exception of apo-12′- and apo-10′-carotenal (Table 3). Thus, current results indicate an incipient saturation of detector response, and the selection of a regression model for individual concentration regimes in correspondence to experimentally measured peak areas seems most advantageous. Although this might be arguable from the statistical point of view, i.e., for MFT results which are not significant, this strategy will reduce the uncertainty calculated as confidence interval when quantifying analytes via regression.

#### Testing for homogeneity of variance

In addition, calibration data were tested for absence of heteroscedasticity, since this would change the uncertainty of analytical results in a concentration dependent manner. Due to its higher sensitivity, quantification of CPs will be done by Orbitrap MS with optional confirmation of concentrations by UV in case analytes are situated in appropriate concentration domains. Therefore, homogeneity of variance was tested for ESI–LTQ–Orbitrap MS by analyzing standard solutions of CPs (including IS) at concentration levels of 0.1 and 5.0 μg/mL by ten injection replicates, respectively. *F*_calc_ was derived by dividing the relative variance of the higher by the relative variance of the lower concentration. In all tested cases, *F*_calc_ was lower than the tabulated *F*_tab_ (=5.34), confirming homogeneity of relative variances over the tested range at a 99 % confidence level (Table 3) [61].

#### Instrumental limit of detection and limit of quantification

LOD and LOQ of BC and CPs were calculated for UV and Orbitrap MS detection according to the ICH guideline Q2(R1) applying the so-called standard deviation approach. Thereby, the standard deviation of signals has to be calculated either from the noise of a blank or from a standard solution or sample containing the respective analytes at low concentrations [59]. Instrumental LOD and LOQ were determined from a combined standard solution containing BC and CPs at 0.025 μg/mL. LOD and LOQ both for the UV and the MS measurement step were calculated to address possible differences and derive applicable minimum concentrations for linearity testing. Baseline fluctuation, i.e. noise, was calculated differently for UV and MS detection. For UV, noise was calculated from the lowest standard concentration considered in linearity testing, i.e., 0.10 μg/mL. Thereby, a retention window considering twice the base peak width on either side of the respective analyte peaks was constructed for determination of the baseline noise. In case of MS detection, noise values given by the software via the signal-to-noise option in the SIM mode were derived for either analyte signal. LOD was calculated as the 3.3-fold of the determined noise divided by the slope of the peak height calibration curve over the low concentration domain for either CP. For LOQ, the 10-fold noise is considered otherwise duplicating the calculation approach for LOD [59]. Since both LOD and LOQ refer to signal heights, calibration curves for peak heights had to be calculated. Due to previous linearity data, considered concentration ranges were selected close to preliminarily estimated LOQs to prevent deviation from linearity. Selected concentration ranges for linearity testing of peak heights comprised 1.00 to 5.00 μg/mL for UV and 0.10 to 1.00 μg/mL for Orbitrap MS. For either concentration range linearity was proven by MFT as outlined in the previous section (data not shown). Calculated LODs and LOQs for UV detection and MS are provided in Table 4. Depending on the respective UV absorbance, LOQs for UV detection were between 52.6 and 889.4 ng/mL. Remarkably, LOQs for Orbitrap MS were between 19.3 and 102.4 ng/L and thus between 5.15 × 10^2^- and 2.60 × 10^4^-fold smaller than their UV counterparts (see Table 4). The low LOD and LOQ realized for BC with Orbitrap MS are related to electrochemical oxidation and formation of radical ions (M^•+^) as described previously [56, 57].

### Validation of SPE

#### Intra-day and inter-day precision of SPE recovery

The SPE protocol pursued for non-volatile CPs and BC refers to [47]. Briefly, the principle of interaction between the phenyl stationary phase employed in SPE and the analytes is mainly based on *π*–*π* interactions. In the current case, the entire 2-mL SPE eluate was applied to UHPLC subsequent to the aforementioned solvent change, but operation with a 1-mL aliquot is applicable as well when employing GC–EI–MS and UHPLC–DAD–ESI–Orbitrap MS in parallel. The intra-day precision of SPE was determined at two concentration levels within the single-laboratory validation, based on five SPE replicates within 1 day, respectively. Model samples for SPE were prepared by spiking MEM with a combined stock solution of CPs and BC to give final concentrations of 1.0 and 0.5 μg/mL for either analyte. Recoveries were between 65.8 and 102.4 % with corresponding CVs ≤4.0 %, respectively (Table 5; *n* = 5). A comparison of the recovery between 1.0 and 0.5 μg/mL (intra-day) by means of a two-sided independent sample *t* test revealed no significant differences. Moving to target cells other than hepatocytes might require the inclusion of fetal calf serum in the cell culture medium. Therefore, SPE recoveries and related intra-day precision have additionally been tested for MEM including 10 % (*v*/*v*) fetal calf serum and addition of BC and CPs to provide final concentrations of 1.0 μg/mL, respectively. Recoveries for BC and CPs were between 64.3 and 99.8 % with CV <5.0 % (*n* = 3). Indeed, recoveries and intermediate precision perfectly corresponded with data derived from fetal calf serum deficient MEM samples for BC and all CPs.

**Table 5:** Intra-day and inter-day SPE recovery for BC and non-volatile CPs

Concentration		Recovery (%)					
		Apo-12′	Apo-10′	Apo-8′	Apo-4′	5,6-Epoxy-BC	BC
1.0 μg/mL	Intra-day (*n* = 5)	74.4	94.6	84.5	84.1	65.8	101.6
	CV%	1.4	1.4	3.1	4.0	2.7	3.6
	Inter-day (*n* = 15)^a^	73.4	93.2	89.5	84.8	66.6	91.1
	CV%	3.4	6.2	5.0	3.8	5.8	4.6
0.5 μg/mL	Intra-day (*n* = 5)	73.2	90.8	84.8	87.2	66.0	102.4
	CV%	3.6	3.7	3.3	2.6	2.0	3.2

^a^The sample size (*n* = 15) refers to three SPEs performed on five consecutive days

Inter-day precision was based on three extractions per day performed over five consecutive days. Spiked model samples were prepared independently on either day. Inter-day recoveries were between 66.6 and 91.1 % (CV ≤6.2 %; *n* = 15). One-factor ANOVA of inter-day recoveries revealed significant differences for apo-10′-, apo-4′, 5,6-epoxy-BC, and BC between individual days, whereas Levene’s test for testing homogeneity of variance was only significant for 5,6-epoxy-BC and BC. The stationary phenyl phase previously applied in the SPE of volatile CPs [47] appears thus equally applicable for the extraction of highly hydrophobic BC and non-volatile target CPs.

#### Specificity

To confirm the method specificity, fresh MEM and MEM after 3 h incubation in primary hepatocyte cultures were subjected to SPE and subsequently analyzed by UHPLC–DAD–ESI–Orbitrap MS. No interfering peaks were observed in UV and MS chromatograms in either case (data not shown). However, with the inclusion of fetal calf serum, a slight blank was revealed that was ≤3 % when compared to peak area of the BC concentration applied in culture treatment (data not shown). This has to be considered when analyzing low BC concentrations in the presence of fetal calf serum.

#### SPE linearity for spiked MEM samples

Preliminary results of BC degradation in cell cultures revealed BC, 5,6-epoxy-BC, and apo-4′ in quantifiable amounts (data not shown). Therefore, SPE linearity was confirmed by spiking MEM with these analytes in appropriate concentrations. Apo-4′ and 5,6-epoxy-BC were quantified by Orbitrap MS. The 100- to 4,000-fold higher concentrations of BC in comparison to these CPs were due to its application in form of the treatment solution and required quantification by DAD. Five concentrations distributed equidistantly over the tested range were analyzed for either compound. In addition, a MEM blank was analyzed. Subsequent to SPE, each concentration was injected in triplicate. Derived calibration curves refer to the mean of the injection replicates, respectively. All calibration curves were linear as proven by MFT. Intercept was not significant for apo-4′and 5,6-epoxy-BC (see Table 6). The addition of 10 % FCS to cell culture media showed neither changes in *t*_R_ of analytes nor in their recovery and recovery precision of SPE. Moreover, the influence on the selectivity was minute, obviously requiring consideration only at greatly reduced concentrations of BC. However, this does not necessarily guarantee the maintenance of regression slopes in UV and MS detection and SPE calibration nor the respective linear ranges confirmed for serum deficient culture media (see Tables 2, 3, and 6). Actually, matrix effects due to addition of fetal calf serum might alter these parameters and additionally change LOD and LOQ. In case of quantification in the presence of fetal calf serum, a comprehensive further validation that is beyond the current scope is mandatory to assure correct concentrations.

**Table 6:** SPE calibration and linearity of MEM samples spiked with BC and selected CPs (*n* = 15)

Analyte	Concentration range (μg/mL)	*y* = *bx* + *a*					Linearity testing MFT^b^
		Slope (= *b*)	ANOVA	Intercept (= *a*)	*p* (for *a*)^a^	*R* ^2^	
Apo-4′	0–0.0125^a^	1,107,257.1	*p* < 0.05*	425.8	>0.05	0.995	Passed
5,6-Epoxy-BC	0–0.50^a^	1,019,107.4	*p* < 0.05*	8450.5	>0.05	0.991	Passed
BC	10–50	6,429,177.1	*p* < 0.05*	191,897.8	<0.05*	0.992	Passed

^a^In either case, a blank MEM was passed over SPE

^b^Mandel’s fitting test at a confidence level of 99.0 %

#### Identification of CPs in treated cell cultures

The validated SPE UHPLC–DAD–ESI–Orbitrap MS method was applied to cell cultures of primary rat hepatocytes. Subsequent to the addition of the BC treatment solution to primary cultures to give a final BC concentration of 10 μM (5360 ng/mL), primary rat hepatocyte cultures were incubated for 3 h and simultaneously subjected to oxidative stress induced by different means. This approach targets to elucidate possible differences in the generated profiles and concentrations of CPs that were produced either directly in the medium and/or in the cells with subsequent secretion. Oxidative stress was either induced by (i) 40 μmol/L DMNQ [21], (ii) 10 μmol/L hydrogen peroxide (H_2_O_2_) [63, 64], or (iii) 10 μmol/L H_2_O_2_ in the presence of 10 μmol/L Fe(II)lactate (the latter simulating the Fenton reaction) [50]. The H_2_O_2_ treatment concentration was selected 10-fold lower than published elsewhere [65], to assure integrity of hepatocytes. The H_2_O_2_ and Fe(II)lactate concentrations were selected equimolar and exerted no cytotoxic effects over the incubation interval [66].

Application of DMNQ was designed as initial treatment since DMNQ induces oxidative stress after its uptake into the cell. This is considered to simulate the cell physiological environment of patients subjected to strong oxidative stress, such as smokers. CPs derived from this treatment and proven not to result from autoxidation in the culture medium can thus be assigned to enzymatic (and possibly non-enzymatic) formation taking place in the cell with subsequent secretion into the medium. The second approach applied H_2_O_2_ as an oxidant agent to promote oxidative stress. In the last approach, hepatocytes were simultaneously incubated with H_2_O_2_ and Fe(II)lactate. According to the Fenton reaction, H_2_O_2_ reacts with Fe^2+^ with consecutive generation of OH^−^ and ^•^OH and Fe^3+^ [50]. In addition, either treatment procedure was also performed (i) by addition of BC in the absence of cells and (ii) in the presence of cells, but without addition of BC. As a further control, (iii) hepatocytes were treated with BC without induction of oxidative stress. All other experimental frame conditions were maintained. To prevent instrumental sample carry-over between analytical runs and thus false positive results, measurement series comprising samples of individual treatments were always completed with blank injections, which provided no signals.

In case cells were treated with BC and subjected to oxidative stress, 5,6-epoxy-BC and apo-4′ were detected in addition to BC, irrespective of the mode of oxidative stress (see Table 7). Remarkably, cell cultures treated with BC but without oxidative stress showed the same profile of CPs, i.e., 5,6-epoxy-BC and apo-4′. However, apo-4′ was only detected in quantifiable amounts in hepatocyte cultures of one rat, but not in cultures of the other in either case. If only BC was added to cell culture medium without cells, 5,6-epoxy-BC was detected as well but not apo-4′, irrespective of the mode of oxidative stress and even in its absence (Table 7). Based on these results, 5,6-epoxy-BC which constitutes the major CP under the selected experimental conditions seems to be formed irrespective of externally induced oxidative stress and the presence of cells. Since no CPs were detected after SPE of MEM solutions spiked with BC, the extraction step can be excluded as a causative source for BC degradation. Instead, the ambient oxygen pressure prevailing during the 3-h incubation period appears sufficient to induce oxidation of BC, forming 5,6-epoxy BC but none of the other target CPs. This provides essential information for the interpretation of adverse effects on cells, since even by exclusive application of BC encountered effects might be related to the formed epoxy variant of BC. In case of apo-4′, the situation is more intriguing. Since this CP was never found in the absence of cells, formation is most likely mediated by cells but depends apparently on their individual response, as not all test animals provided this CP. Altogether, the application of the validated SPE UHPLC–DAD–ESI–Orbitrap MS method allowed for a deeper insight into CP profiles and quantification of individual target CPs formed in the course of in vitro experiments. This is an essential prerequisite for the interpretation of adverse cell effects when trying to reveal possible mechanisms attempting to mimic the BC paradox in probationers suffering from oxidative stress, such as smokers and asbestos workers.

**Table 7:** Non-volatile CPs of BC identified in MEM without and with cells after addition of 10 μmol/L BC under control conditions and oxidative stress induced by different chemical means (DMNQ, H_2_O_2_, and Fe/H_2_O_2_), respectively

		Apo-4′^a^ (μg/mL)	5,6-Epoxy-BC (μg/mL)	BC (μg/mL)
Treatment in MEM without cells				
BC control	Mean	n.d.	0.33	4.79
	*s*		0.21	0.24
BC DMNQ	Mean	n.d.	0.17	4.99
	*s*		0.03	0.10
BC H_2_O_2_	Mean	n.d.	0.17	5.14
	*s*		0.02	0.17
BC Fe/H_2_O_2_	Mean	n.d.	0.16	4.90
	*s*		0.04	0.29
Treatment in MEM with primary hepatocytes				
Control BC	Mean	0.005	0.15	4.65
	*s*	0.007	0.04	0.57
BC DMNQ	Mean	0.008	0.11	4.67
	*s*	0.009	0.03	0.33
BC H_2_O_2_	Mean	0.006	0.10	4.64
	*s*	0.008	0.02	0.58
BC Fe/H_2_O_2_	Mean	0.006	0.12	4.52
	*s*	0.007	0.03	0.16

*n.d.* not determined

^a^Apo-4′ was only detected in primary cultures derived from one rat (for details, see text)

## Conclusion

For the first time this work presents a fast UHPLC method employing sub-2 μm core–shell particles for separation of BC and related long-chain CPs. UHPLC hyphenation to DAD and—via electrospray ionization—to LTQ–Orbitrap MS allows the identification and quantification of these analytes in cell culture media. Preceding offline SPE as well as the applied internal standard are shared with a GC–EI–MS method for volatile CPs previously published by our group [47]. This permits a simultaneous SPE of volatile and non-volatile CPs and BC under identical extraction conditions and thus a more comprehensive investigation of BC degradation in in vitro systems subjected to oxidative stress. The method was validated for BC and selected CPs which are currently considered most relevant for adverse effects observed in risk groups of BC intervention studies. Therefore, intra-day and inter-day precision for peak areas and retention times, linearity of detector responses, LOD and LOQ, as well as intra- and inter-day SPE recoveries were determined. LOQs between 19.3 and 102.4 ng/L for Orbitrap MS allowed for a trace detection of CPs. The profiling and identification of CPs by LTQ–Orbitrap MS with high mass accuracy as well as their quantification in primary cell cultures is essential to relate observed genotoxic effects with prevailing ensembles of CPs. When culture media were incubated with BC, 5,6-epoxy-BC was quantified after 3 h irrespective of oxidative stress. Apparently the formation occurs during the incubation by ambient oxygen. The observed concentration of this CP was equivalent in the presence and absence of primary rat hepatocytes. However, within a primary hepatocyte population derived from one rat, an early long-chain CP, i.e., apo-4′-carotenal, was generated under oxidative stress in all tested cultures, but not in a second biological replicate. This might indicate individual degradation kinetics over the investigated 3-h incubation. Since the method has proven its applicability for cell culture media, it will provide an essential input for an improved interpretation of in vitro models and contribute to a future deciphering of the BC paradox by testing various BC concentrations with increased biological replicates.

## Abbreviations

ACN: Acetonitrile
BC: β-Carotene
CPs: Cleavage products of β-carotene
CV: Coefficient of variation
DAD: Diode array detection
DMNQ: 2,3-Dimethoxy-1,4-naphthoquinone
EICC: Extracted ion current chromatogram
GC–EI–MS: Gas chromatography–electron ionization–mass spectrometry
IS: Internal standard
LOD: Limit of detection
LOQ: Limit of quantification
LTQ: Linear quadrupole ion trap
MEM: Minimum essential medium Eagle
MFT: Mandel’s fitting test
MS: Mass spectrometry
SPE: Solid-phase extraction
TFA: Trifluoroacetic acid
THF: Tetrahydrofuran
*t*_R_: Retention time
UHPLC: Ultrahigh-performance liquid chromatography

## References

[1] Peto R, Doll R, Buckley JD, Sporn MB (1981). Can dietary beta-carotene materially reduce human cancer rates?. Nature.

[2] Lee CM, Boileau AC, Boileau TWM, Williams AW, Swanson KS, Heintz KA, Erdman JW (1999). Review of animal models in carotenoid research. J Nutr.

[3] Ferrari CKB (2007). Functional foods and physical activities in health promotion of aging people. Maturitas.

[4] Burton G, Ingold K (1984). beta-Carotene: an unusual type of lipid antioxidant. Science.

[5] Paiva SAR, Russell RM (1999). B-Carotene and other carotenoids as antioxidants. J Am Coll Nutr.

[6] Barua AB (1999). Intestinal absorption of epoxy-beta-carotenes by humans. Biochem J.

[7] Breithaupt DE (2004). Simultaneous HPLC determination of carotenoids used as food coloring additives: applicability of accelerated solvent extraction. Food Chem.

[8] Rietjens IMCM, Boersma MG, de Haan L,, Spenkelink B, Awad HM, Cnubben NHP, van Zanden JJ, Hvd W, Alink GM, Koeman JH (2002) The pro-oxidant chemistry of the natural antioxidants vitamin C, vitamin E, carotenoids and flavonoids. Environ Toxicol Pharmacol 11(3–4):321–333 10.1016/s1382-6689(02)00003-0 21782615

[9] Paolini M, Abdel-Rahman SZ, Sapone A, Pedulli GF, Perocco P, Cantelli-Forti G, Legator MS (2003). β-Carotene: a cancer chemopreventive agent or a co-carcinogen?. Mutat Res.

[10] Siems W, Salerno C, Crifó C, Sommerburg O, Wiswedel I, Yoshikawa T (2009). Forum of nutrition. Food factors for health nutrition.

[11] Albanes D, Heinonen OP, Taylor PR, Virtamo J, Edwards BK, Rautalahti M, Hartman AM, Palmgren J, Freedman LS, Haapakoski J, Barrett MJ, Pietinen P, Malila N, Tala E, Liippo K, Salomaa E-R, Tangrea JA, Teppo L, Askin FB, Taskinen E, Erozan Y, Greenwald P, Huttunen JK (1996). α-Tocopherol and β-carotene supplements and lung cancer incidence in the alpha-tocopherol, beta-carotene cancer prevention study: effects of base-line characteristics and study compliance. J Natl Cancer Inst.

[12] Omenn GS, Goodman GE, Thornquist MD, Balmes J, Cullen MR, Glass A, Keogh JP, Meyskens FL, Valanis B, Williams JH, Barnhart S, Cherniack MG, Brodkin CA, Hammar S (1996). Risk factors for lung cancer and for intervention effects in CARET, the beta-carotene and retinol efficacy trial. J Natl Cancer Inst.

[13] Omenn GS, Goodman GE, Thornquist MD, Balmes J, Cullen MR, Glass A, Keogh JP, Meyskens FL, Valanis B, Williams JH, Barnhart S, Hammar S (1996). Effects of a combination of beta carotene and vitamin A on lung cancer and cardiovascular disease. N Engl J of Med.

[14] Hennekens CH, Buring JE, Manson JE, Stampfer M, Rosner B, Cook NR, Belanger C, LaMotte F, Gaziano JM, Ridker PM, Willett W, Peto R (1996). Lack of effect of long-term supplementation with beta carotene on the incidence of malignant neoplasms and cardiovascular disease. N Engl J Med.

[15] Blot WJ, Li J-Y, Taylor PR, Guo W, Dawsey S, Wang G-Q, Yang CS, Zheng S-F, Gail M, Li G-Y, Yu Y, Liu B-q, Tangrea J, Sun Y-h, Liu F, Fraumeni JF, Zhang Y-H, Li B (1993). Nutrition intervention trials in Linxian, China: supplementation with specific vitamin/mineral combinations, cancer incidence, and disease-specific mortality in the general population. J Natl Cancer Inst.

[16] Russell RM (2002). β-Carotene and lung cancer. Pure Appl Chem.

[17] Liu C, Wang X-D, Bronson RT, Smith DE, Krinsky NI, Russell RM (2000). Effects of physiological versus pharmacological β-carotene supplementation on cell proliferation and histopathological changes in the lungs of cigarette smoke-exposed ferrets. Carcinogenesis.

[18] Arora A, Willhite CA, Liebler DC (2001). Interactions of β-carotene and cigarette smoke in human bronchial epithelial cells. Carcinogenesis.

[19] Mein JR, Dolnikowski GG, Ernst H, Russell RM, Wang X-D (2011). Enzymatic formation of apo-carotenoids from the xanthophyll carotenoids lutein, zeaxanthin and β-cryptoxanthin by ferret carotene-9′,10′-monooxygenase. Arch Biochem Biophys.

[20] Siems W, Wiswedel I, Salerno C, Crifò C, Augustin W, Schild L, Langhans C-D, Sommerburg O (2005). β-Carotene breakdown products may impair mitochondrial functions—potential side effects of high-dose [beta]-carotene supplementation. J Nutr Biochem.

[21] Alija AJ, Bresgen N, Sommerburg O, Langhans CD, Siems W, Eckl PM (2006). β-Carotene breakdown products enhance genotoxic effects of oxidative stress in primary rat hepatocytes. Carcinogenesis.

[22] Alija AJ, Bresgen N, Sommerburg O, Siems W, Eckl PM (2004). Cytotoxic and genotoxic effects of β-carotene breakdown products on primary rat hepatocytes. Carcinogenesis.

[23] Sugiyama S, Okada Y, Sukhova GK, Virmani R, Heinecke JW, Libby P (2001). Macrophage myeloperoxidase regulation by granulocyte macrophage colony-stimulating factor in human atherosclerosis and implications in acute coronary syndromes. American J Pathol.

[24] Handelman GJ, van Kuijk FJGM, Chatterjee A, Krinsky NI (1991). Characterization of products formed during the autoxidation of β-carotene. Free Radical Biol Med.

[25] Sommerburg O, Langhans C-D, Arnhold J, Leichsenring M, Salerno C, Crifò C, Hoffmann GF, Debatin K-M, Siems WG (2003). β-Carotene cleavage products after oxidation mediated by hypochlorous acid—a model for neutrophil-derived degradation. Free Radical Biol Med.

[26] Wang X-D, Tang G-W, Fox JG, Krinsky NI, Russell RM (1991). Enzymatic conversion of β-carotene into β-apo-carotenals and retinoids by human, monkey, ferret, and rat tissues. Arch Biochem Biophys.

[27] Shmarakov I, Fleshman MK, D’Ambrosio DN, Piantedosi R, Riedl KM, Schwartz SJ, Curley RW, Von Lintig J, Rubin LP, Harrison EH, Blaner WS (2010). Hepatic stellate cells are an important cellular site for β-carotene conversion to retinoid. Arch Biochem Biophys.

[28] Barua AB, Olson JA (1998). Reversed-phase gradient high-performance liquid chromatographic procedure for simultaneous analysis of very polar to nonpolar retinoids, carotenoids and tocopherols in animal and plant samples. J of Chromatogr B: Biomed Sci Appl.

[29] Thibeault D, Su H, MacNamara E, Schipper HM (2009). Isocratic rapid liquid chromatographic method for simultaneous determination of carotenoids, retinol, and tocopherols in human serum. J Chromatogr B.

[30] Rajendran V, Pu YS, Chen BH (2005). An improved HPLC method for determination of carotenoids in human serum. J Chromatogr B.

[31] Nakagawa K, Kiko T, Hatade K, Asai A, Kimura F, Sookwong P, Tsuduki T, Arai H, Miyazawa T (2008). Development of a high-performance liquid chromatography-based assay for carotenoids in human red blood cells: application to clinical studies. Anal Biochem.

[32] Lacker T, Strohschein S, Albert K (1999). Separation and identification of various carotenoids by c30 reversed-phase high-performance liquid chromatography coupled to UV and atmospheric pressure chemical ionization mass spectrometric detection. J Chromatogr A.

[33] Chauveau-Duriot B, Doreau M, Nozière P, Graulet B (2010). Simultaneous quantification of carotenoids, retinol, and tocopherols in forages, bovine plasma, and milk: validation of a novel UPLC method. Anal Bioanal Chem.

[34] Rodríguez AM, Sastre S, Ribot J, Palou A (2005). beta-Carotene uptake and metabolism in human lung bronchial epithelial cultured cells depending on delivery vehicle. Biochim Biophys Acta (BBA)—Mol Basis Dis.

[35] Hagiwara T, Yasuno T, Funayama K, Suzuki S (1998). Determination of lycopene, α-carotene and β-carotene in serum by liquid chromatography-atmospheric pressure chemical ionization mass spectrometry with selected-ion monitoring. J Chromatogr B.

[36] Rodriguez EB, Rodriguez-Amaya DB (2007). Formation of apocarotenals and epoxycarotenoids from β-carotene by chemical reactions and by autoxidation in model systems and processed foods. Food Chem.

[37] Ho CC, de Moura FF, Kim S-H, Clifford AJ (2007). Excentral cleavage of β-carotene in vivo in a healthy man. Am J of Clin Nutr.

[38] Fekete S, Ganzler K, Fekete J (2011). Efficiency of the new sub-2 μm core–shell (Kinetex^TM^) column in practice, applied for small and large molecule separation. J Pharm Biomed Anal.

[39] Fekete S, Oláh E, Fekete J (2012). Fast liquid chromatography: the domination of core–shell and very fine particles. J Chromatogr A.

[40] Guillarme D, Ruta J, Rudaz S, Veuthey J-L (2010). New trends in fast and high-resolution liquid chromatography: a critical comparison of existing approaches. Anal Bioanal Chem.

[41] Ruta J, Zurlino D, Grivel C, Heinisch S, Veuthey J-L, Guillarme D (2012). Evaluation of columns packed with shell particles with compounds of pharmaceutical interest. J Chromatogr A.

[42] Oláh E, Fekete S, Fekete J, Ganzler K (2010). Comparative study of new shell-type, sub-2 μm fully porous and monolith stationary phases, focusing on mass-transfer resistance. J Chromatogr A.

[43] Gritti F, Leonardis I, Shock D, Stevenson P, Shalliker A, Guiochon G (2010). Performance of columns packed with the new shell particles, Kinetex-C_18_. J Chromatogr A.

[44] Hogenboom AC, van Leerdam JA, de Voogt P (2009). Accurate mass screening and identification of emerging contaminants in environmental samples by liquid chromatography–hybrid linear ion trap Orbitrap mass spectrometry. J Chromatogr A.

[45] Perry RH, Hu Q, Salazar GA, Cooks RG, Noll RJ (2009). Rephasing ion packets in the Orbitrap mass analyzer to improve resolution and peak shape. J Am Soc Mass Spectrom.

[46] van der Heeft E, Bolck YJC, Beumer B, Nijrolder AWJM, Stolker AAM, Nielen MWF (2009). Full-scan accurate mass selectivity of ultra-performance liquid chromatography combined with time-of-flight and Orbitrap mass spectrometry in hormone and veterinary drug residue analysis. J Am Soc Mass Spectrom.

[47] Martano G, Vogl C, Bojaxhi E, Bresgen N, Eckl P, Stutz H (2011). Solid-phase extraction and GC–MS analysis of potentially genotoxic cleavage products of β-carotene in primary cell cultures. Anal Bioanal Chem.

[48] Eckl PM, Whitcomb WR, Michalopoulos G, Jirtle RL (1987). Effects of EGF and calcium on adult parenchymal hepatocyte proliferation. J Cell Physiol.

[49] Eckl P, Bresgen N (2003). The cultured primary hepatocyte and its application in toxicology. J Appl Biomed.

[50] Agil A, Fuller C, Jialal I (1995). Susceptibility of plasma to ferrous iron/hydrogen peroxide-mediated oxidation: demonstration of a possible Fenton reaction. Clin Chem.

[51] Citová I, Havlíková L, Urbánek L, Solichová D, Nováková L, Solich P (2007). Comparison of a novel ultra-performance liquid chromatographic method for determination of retinol and α-tocopherol in human serum with conventional HPLC using monolithic and particulate columns. Anal Bioanal Chem.

[52] Fleshman MK, Lester GE, Riedl KM, Kopec RE, Narayanasamy S, Curley RW, Schwartz SJ, Harrison EH (2011). Carotene and novel apocarotenoid concentrations in orange-fleshed *Cucumis melo* melons: determinations of β-carotene bioaccessibility and bioavailability. J Agric Food Chem.

[53] Staub A, Zurlino D, Rudaz S, Veuthey J-L, Guillarme D (2011). Analysis of peptides and proteins using sub-2 μm fully porous and sub 3 μm shell particles. J Chromatogr A.

[54] Feltl L, Pacakova V, Stulik K, Volka K (2005). Reliability of carotenoid analyses: a review. Curr Anal Chem.

[55] Wu Z, Robinson DS, Hughes RK, Casey R, Hardy D, West SI (1999). Co-oxidation of β-carotene catalyzed by soybean and recombinant pea lipoxygenases. J Agric Food Chem.

[56] van Breemen RB (1995). Electrospray liquid chromatography–mass spectrometry of carotenoids. Anal Chem.

[57] Vessecchi R, Crotti AEM, Guaratini T, Colepicolo P, Galembeck SE, Lopes NP (2007). Radical ion generation processes of organic compounds in electrospray ionization mass spectrometry. Mini-Rev Org Chem.

[58] Van Berkel GJ, Zhou F (1994). Chemical electron-transfer reactions in electrospray mass spectrometry: effective oxidation potentials of electron-transfer reagents in methylene chloride. Anal Chem.

[59] ICH (2005). Validation of analytical procedures: text and methodology Q2(R1).

[60] Thompson M, Ellison SLR, Wood R (2002). Harmonized guidelines for single-laboratory validation of methods of analysis (IUPAC technical report). Pure Appl Chem.

[61] Funk W, Dammann V, Donnevert G (2007) Quality assurance in analytical chemistry: applications in environmental, food and materials analysis, biotechnology, and medical engineering, 2nd revised edn. Wiley-VCH, Weinheim

[62] Einax J, Reichenbächer M (2006). Solution to quality assurance challenge 2. Anal Bioanal Chem.

[63] Halliwell B, Clement MV, Long LH (2000). Hydrogen peroxide in the human body. FEBS Lett.

[64] Jonas SK, Riley PA, Willson RL (1989). Hydrogen peroxide cytotoxicity. Low-temperature enhancement by ascorbate or reduced lipoate. Biochem J.

[65] van Helden YGJ, Keijer J, Heil SG, Picó C, Palou A, Oliver P, Munnia A, Briedé JJ, Peluso M, Franssen-van Hal NL, van Schooten FJ, Godschalk RWL (2009). β-carotene affects oxidative stress-related DNA damage in lung epithelial cells and in ferret lung. Carcinogenesis.

[66] Glei M, Latunde-Dada GO, Klinder A, Becker TW, Hermann U, Voigt K, Pool-Zobel BL (2002). Iron-overload induces oxidative DNA damage in the human colon carcinoma cell line HT29 clone 19A. Mutat Res Genet Toxicol Environ Mutagen.

